# Anthropometry and Menarcheal Status of Adolescent Nigerian Urban Senior Secondary School Girls

**DOI:** 10.5812/ijem.8052

**Published:** 2013-04-01

**Authors:** Alphonsus Ndidi Onyiriuka, Eruke Elizabeth Egbagbe

**Affiliations:** 1 Department of Medicine, University of Benin Teaching Hospital, Benin City, Nigeria

**Keywords:** Body Mass Index, Height, weight, Ponderal Index, Menarche

## Abstract

**Background:**

Age at menarche is a significant indicator of growth and sexual maturation in girls. During adolescence, anthropometry provides a tool for monitoring and evaluating the hormone-mediated changes in growth and reproductive maturation.

**Objectives:**

We aimed to examine the anthropometric status of pre- and post-menarcheal Nigerian adolescent girls attending senior secondary schools.

**Materials and Methods:**

In this school-based cross-sectional survey, a pre-tested structured self-administered questionnaire was set for obtaining the socio-demographic data (age at menarche, number of siblings, occupation and educational attainment of their parents, etc.), while the anthropometric status data was obtained by direct measurement of weight and height. The body mass index (BMI) and the ponderal index (PI) of each participant were computed from their respective weight and height values. The study was designed to include all the students in the two schools that were randomly selected. The anthropometric indices of pre- and post-menarcheal girls were compared.

**Results:**

Out of a total population of 2,166 students, 2,159 (99.7%) participated but 9 questionnaires were incompletely filled and were rejected, leaving 2,150 (510 were pre-menarcheal and 1,640 were post- menarcheal) for further analysis. The mean menarcheal age was 13.44 ± 1.32 years (95% Confidence Interval (CI) = 13.38-13.5). Girls from families with high socio-economic status (SES) attained menarche 8.0 and 9.0 months earlier than their counterparts from families with middle and low SES respectively. Girls from small-size families had a significantly lower menarcheal age than their counterparts from large-size families. A comparison of the anthropometric indices of pre- and post-menarcheal girls showed: weight, 41.1 ± 6.3 kg (95% CI = 40.6-41.6) vs 47.6 ± 7.2 kg (95% CI = 47.3-47.9), P < 0.001; height, 146.2± 5.5 cm (95% CI = 145.7-146.7) vs 153.6 ± 9.9 cm (95% CI = 153.1-154.1), P < 0.001; BMI, 16.4 ± 1.9 (95% CI = 16.2-16.6) vs 18.8 ± 1.6 (95% CI = 18.7-18.9), P < 0.001; and PI, 45.1 ± 1.7 (95% CI = 45.0-45.2) vs 44.6 ± 1.4 (95% CI = 44.5-44.7), P < 0.01.

**Conclusions:**

Post-menarcheal girls were significantly taller and heavier with a higher BMI than their pre-menarcheal counterparts, but the pre-menarcheal girls possessed a better linear body as reflected by the PI.

## 1. Background

The adolescent age group constitutes a large segment of the population of any nation. For instance, in Nigeria adolescents account for approximately 23% of the total population (1). Menarche is the first menstrual bleeding in the life of a female and represents a major landmark event in the life of an adolescent girl. This explains why it is the most accurately recalled indicator of puberty. In addition, menarche is the last in the series of events involved in the process of normal pubertal development. Increasing our knowledge about the various factors regulating the onset age of menarche has the potential of improving our understanding of female reproductive health, ultimately promoting the health of women and their offspring. For instance, the age at menarche is not only a reflection of onset of ovarian function, but also, a predictor of ovulatory frequency (2). The age at which an individual attains menarche depends not only on genetic potential, but also on health status, which is in turn influenced by environmental factors, such as general standards of living and nutrition. The report of some studies revealed that socio-economic status of the family as well as family size influenced the menarcheal age of girls from these families (3-5).

Anthropometric measurements are an important, widely applicable, non-invasive, and inexpensive technique for assessing body size, proportions, and composition. With regard to the adolescent, anthropometry is particularly important because it acts as a tool for monitoring and evaluating the hormone-mediated changes in growth and reproductive maturation during this phase of life. In a study at Ile Ife, Nigeria, Dare et al. (5) reported the existence of considerable variations in the weight, height and, body mass index (BMI) of adolescent girls who attained menarche. The reports of several studies separately indicated that adolescent girls who attained menarche demonstrated a significantly higher weight, height, and BMI than their peers who have not attained menarche (5-7). The ponderal index (PI) has been used as a tool for studying the physique of adolescent girls and measuring the linearity of the body (8) . PI is commonly used in Pediatrics because the resulting values are consistent across varying heights (9). Various studies have established a link between PI and age at the onset of menarche (8, 10, 11). Among the various anthropometric measurements, BMI has been found to be the most relevant to the onset of menarche (3, 7). Therefore, in this context BMI may be useful in the assessment of patients with delayed onset of menarche. Given that in both developed and developing countries, reports from various studies revealed a trend towards a reduction in the average age of menarche, where this was attributed to the improvement in the standard of living and nutrition (12-16). This leads to a surmise that this scenario would be reflected in the anthropometric indices of the adolescent school girls.

Although there are several studies conducted decades ago in Nigeria that have examined the age of onset of menarche, only a few have examined the anthropometry and menarcheal status among the students (17). In Nigeria, reports on comparative nutritional status based on anthropometric indices between pre-and post-menarcheal girls are very scarce. To the best of our knowledge, there is no Nigerian study that has examined the correlation between PI and menarcheal status, indicating that anthropometry in relation to menarcheal status has not received sufficient attention in Nigeria.

## 2. Objectives

These factors prompted us to assess the anthropometric status of pre- and post-menarcheal schoolgirls. The present study, therefore, sought to examine the anthropometric status of pre- and post-menstrual adolescent girls attending senior secondary school in Benin City, Nigeria.

## 3. Materials and Methods

This cross-sectional survey was conducted in two public girls' secondary schools in Oredo's Local Governmental Area (OLGA), Edo State, Nigeria. In this LGA, there are nine public secondary schools comprising 4 females-only, 3 co-educational, and 2 males-only (18). The study was approved by the school authorities. The teachers distributed parental consent forms to parents via the students asking for permission for the child to participate in the study. Of the four girls’ secondary schools, two were randomly selected by ballot. The total population of students in both schools selected was 1,394 in school A and 772 in school B, summing up all together into 2,166 which was the target study population. The survey was designed to include all the students in both schools (schools A and B). The principal of each of the two schools introduced the authors during the morning assembly. During data collection between October and November of 2011, the students were informed about the relevance of the study and the need to accurately fill the questionnaire without including their names and that their participation was voluntary. Information sought in the questionnaire included: date of birth, date (month and year) of onset of first menstrual bleeding, state of origin, educational attainment, occupation of both parents or guardians, and family size (number of siblings). The menarcheal age was verified from the mother or close relative of each respondent via a telephone call to ensure reliability. The socio-economic status of the parents was determined using the classification suggested by Ogunlesi et al (19). This was analyzed via combining the highest educational attainment, occupation, and income of the parents (based on the mean income of each educational qualification and occupation). In this Social Classification System, classes I and II represent high social class, class III represents middle social class, while classes IV and V represent low social class. In this way, the girls were categorized into high, middle, and low socio-economic groups. The family size was categorized into small size (no sibling or one or 2 siblings); medium size (3 or 4 siblings); large size (5 or more siblings). All participants had a significant acute illness in the past six months preceding the study. Students known to have chronic illnesses such as sickle cell anaemia and diabetes mellitus were excluded.

All the height and weight measurements were performed by one of the authors (ANO), using the standard anthropometric methods of the International Society for the Advancement of Kinanthropometry (ISAK) (20).The height of each of the participants was measured to the nearest 0.1 cm with the subject being bare feet and standing upright against a wall-mounted stadiometer. The body weight of each of the participants was measured to the nearest 0.5 kg, using a balance weighing scale with the subject wearing light cotton school uniform. The body mass index (BMI) of each of the participants was computed from the ratio of the mass in kg to the square of the height in m, [BMI= mass (kg)/height (m^2^)]. The ponderal index (PI) was computed from the ratio of the height in cm to the cube root of the mass in kg [(PI=height (cm)/mass (kg)^1^^/3^)]. The data was analyzed using SPSS (Statistical Package for Social Sciences), version 12.0. The Student t- test was used in comparing the mean of the anthropometric indices of pre- and post-menarcheal schoolgirls. The level of significance was set at P < 0.05.

## 4. Results 

At the time of this survey, a total of 2,166 female students (1,394 in school A and 772 in school B) were attending the two public girls’ secondary schools in the LGA (randomly selected by ballot). Seven students (5 from school A and 2 from school B) declined to participate. The response rates were 99.4% in school A, 99.7% in school B, and 99.7% overall. The questionnaires of 9 students were excluded from the analysis because they were incompletely filled, thereby leaving a total of 2,150 questionnaires (respondents) for data analysis. Students in both schools had similar socio-demographic characteristics, thus further analysis of data was carried out for the combined group of students.

Among the 2,150 respondents, 1,640 (76.3%) have attained menarche, while the remaining 510 (23.7%) have not. The mean age at menarche was 13.44 ± 1.32 years (95% CI = 13.38-13.50). Thirty one (1.9%) of the 1,640 girls attained menarche before the age of 12 years (early menarche). As depicted in Table 1, the mean age at menarche was significantly lower in high socio-economic group compared to the low socio-economic group. Girls from families with high SES attained menarche 8.0 and 9.0 months earlier than their counterparts from families with middle and low SES respectively. Similarly, girls from small-size families had a significantly lower menarcheal age than their counterparts from large- size families (Table 1).

**Table 1.** Socio-economic Status (SES), Family Size, and Mean Menarcheal of Participants

Socioeconomic Status (SES)	No (%)	Mean menarcheal Age, y	95% ConfidenceInterval	t-statistic (P value)
**High SES**	205 (12.5)	12.78 ± 1.21^ a^	12.61-12.95	a vs b = 4.41 (<0.01)
**Middle SES**	556 (33.9)	13.42 ± 1.18^ b^	13.32-13.52	b vs c = 2.08 (> 0.05)
**Low SES**	879 (53.6)	13.56 ± 1.29^ c^	13.42-13.59	a vs c = 7.95 (< 0.001)
**Total**	1640 (100.0)	13.44 ± 1.32^ d^	13.38-13.50	
**Family size**				
**Small size**	221 (13.5)	13.09 ± 1.31^ e^	12.91-13.26	d vs f:t = 4.82 (< 0.01)
**Medium size**	1063 (64.8)	13.41 ± 1.28^ f^	13.33-13.49	d vs e:t = 3.32 (<0.05)
**Large size**	356 (21.7)	13.64 ± 1.37	13.50-13.78	b vs f:t = 2.79 (<0.05)
**Total**	1640 (100.0)	13.44 ± 1.32	13.38-13.50	

Eight (0.5%) of the post-menarcheal girls were the only child in their families and their mean menarcheal age was 12.80 ± 1.11 years (95% CI-=12.03-13.57). The mean weight, height, and BMI of pre- and post-menarcheal adolescent school girls are compared in Table 2, and all the three variables were found to be significantly higher in post-menarcheal girls. As shown in Table 2, pre-menarcheal girls had a significantly higher PI than their post-menarcheal counterparts. The menarcheal age did not differ in relation to state of origin.

**Table 2.** Comparison of Mean Weight, Height, Body Mass Index, and Ponderal Index of Pre- and Post-Menarcheal School Girls

Anthropometric Variables	Pre-Menarche (n = 510), Mean ± SD a (95% CI) a	Post-Menarche (n = 1640), Mean ± SD (95% CI)	t- Statistic(P value)
**Weight, kg **	41.1 ± 6.3 (40.6-41.6)	47.6 ± 7.2 ( 47.3-47.9)	19.65 (<0.001)
**Height, cm **	146-2 ± 5.5 (145.7-146.7)	153.6 ± 9.9 ( 153.1-154.1)	21.44 (0.001)
**BMI, kg/m ^2^**	16.4 ± 1.9 (16.2-16.6)	18.8 ± 1.6 (18.7-18.9)	25.82( <0.001)
**Ponderal index, kg/cm ^3^**	45.1 ± 1.7 (45.0-45.2)	44.6 ± 1.4 (44.5-44.7)	6.04 (<0.01)

^a^Abbreviations: BMI, body mass index; SD=standard deviation; CI, confidence interval

## 5. Discussion

Data from the present study indicate that girls who have attained menarche are significantly heavier, taller, and with higherBMI than their counterparts who are yet to attain menarche. This is not surprising as it is consistent with the reports of earlier studies from both developed and developing countries (3, 5, 16, 17). A high BMI, a heuristic proxy for human adiposity, has been shown to predict earlier onset of menarche (8, 21).The increased body fat reflected by higher BMIin post-menarcheal girls may be related to leptin (a fat-derived protein) which stimulates secretion of gonadotropin-releasing hormone (GnRH) by the hypothalamus, resulting in stimulation of the pituitary-ovarian axis which in turn stimulates pubertal surge of gonadotropin (22). Some studies suggest that the age at menarche is related more to body fat distribution. In this regard, higher gluteofemoral adiposity has been associated with a lower menarcheal age (23).

In the present study, pre-menarcheal girls exhibited a significantly higher mean PI (a measure of linearity of physique) than their post-menarcheal peers. The normative value used in interpreting the PI was based on figures obtained from Indian population because there is none for Nigerian population (8). A higher PI indicates a linear body build (i.e., less weight for height). This finding corroborates the report of an earlier study (10) and suggests that body size exerts some influence on the timing of sexual maturation and that linearity of physique is associated with late maturation. The clinical implication is that a more linear physique (identified by a high mean PI) is associated with a delay in the attainment of menarche. Girls who attained menarche at a later age, tend to achieve a greater height as adults than girls who attained menarche at an earlier age (24, 25). This relationship is explained by the earlier fusion of the growth plate in girls who attained menarche early due to an increase in production of ovarian estrogens associated with menarche (24). The practical implication is that girls who had an early menarcheal age are more likely to have short stature as adults with an attending psychological impact on the long run.

The mean age at menarche (13.44 years) observed in the present study is comparable to 13.43 years reported in 2007 among urban school girls in Port Harcourt, Nigeria (26), but lower than 13.98 years reported in 1994 from Ile Ife, Nigeria (3).This finding might be a reflection of a trend towards a reduction in menarcheal age in Nigeria. Other recent studies in Nigeria have also alluded to the reduction in age at menarche (17). It must be noted that the methods of collecting and analyzing data vary from one study to another, indicating the need to exercise caution when comparing age at menarche in different studies. Consistent with an earlier study in Benin City, (27) nearly 2% of the girls attained menarche before the age of 12 years (early menarche) in the present study. This is worrisome because early menarche has been identified as a risk factor for teenage depression,(28) insulin resistance, (29) and breast cancer in adulthood (30).

The socio-economic status (SES) of the parents had a significant influence on the menarcheal age of their daughters in the present study. Girls from families with high SES attained menarche at a younger age than their counterparts from families with low SES. This finding is consistent with the reports of other studies (3, 27). In contrast, a study from Northern Nigeria did not find any difference in the mean age at menarche in relation to socioeconomic status (31). They attributed their finding to thevery skewed distribution of the social classes. For example, 80.9% and 91.7% of their urban and rural schoolgirls, respectively, came from low SES family. Similarly, girls from small-size families attained menarche at a younger age than their counterparts from large-size families. A possible explanation is that girls from families of high SES and small-size families are exposed to a better standard of living including nutrition. A previous study has reported a similar finding (4). Family size may exert its effect on age at menarche through concealed poverty because the larger the family size the lower the income per capita. In families with low SES, food security is precarious with the resultant inadequate nutrition and stress, a situation that may be compounded by large family size.

Some limitations of the present study need to be considered. The findings are limited by the sampling location since the participants were derived from schoolgirls in only one LGA in an urban area. The basic nature of the study is associated with recall bias, and despite the limitations, this study gave an insight into the anthropometric status of pre- and post-menarcheal school girls in this locality. The strength of the study lies in the large study population which allows for meaningful conclusions. It is therefore suggested that the scope of future studies on the subject should be broadened to include secondary schools in other LGAs of the state as well as adolescents in the rural areas.

In conclusion, although post-menarcheal girls were significantly taller and heavier with a higher BMI than their pre-menarcheal counterparts, the pre-menarcheal girls possessed a better linear body physique.

## References

[1] (1991). National Population Commission, Abuja, Nigeria.

[2] Bernstein L (2002). Epidemiology of endocrine-related risk factors for breast cancer.. J Mammary Gland Biol Neoplasia..

[3] Abioye-Kuteyi EA, Ojofeitimi EO, Aina OI, Kio F, Aluko Y, Mosuro O (1997). The influence of socioeconomic and nutritional status on menarche in Nigerian school girls.. Nutr Health..

[4] Cameron N, Nagdee I (1996). Menarcheal age in two generations of South African Indians.. Ann Hum Biol..

[5] Dare FO, Ogunniyi SO, Makinde OO (1992). Biosocial factors affecting menarche in a mixed Nigerian population.. Cent Afr J Med..

[6] Hesketh T, Ding QJ, Tomkins A (2002). Growth status and menarche in urban and rural China.. Ann Hum Biol..

[7] Lee JM, Appugliese D, Kaciroti N, Corwyn RF, Bradley RH, Lumeng JC (2007). Weight status in young girls and the onset of puberty.. Pediatrics..

[8] Talwar I, Kansal A, Jindal P (2012). Growth pattern and menarcheal status in relation to body size and physique among adolescent rural girls of Pinjore-Nalagerb dun Valley.. Hum Biol Rev..

[9] Ditmier Lawrence F (2006). New Developments in Obesity Research..

[10] Banik Sudip Datta (2011). Evaluation of health status of pre-menarcheal and post-menarcheal girls by Rohrer index in Purulia, West Bengal.. Journal of Public Health and Epidemiology..

[11] Elizondo S (1992). Age at menarche: its relation to linear and ponderal growth.. Ann Hum Biol..

[12] Anderson SE, Dallal GE, Must A (2003). Relative weight and race influence average age at menarche: results from two nationally representative surveys of US girls studied 25 years apart.. Pediatrics..

[13] Bhadra M, Mukhopadhyay A, Bose KS (2001). Body mass index, regional adiposity and central body fat distribution among Bengalee Hindu girls: a comparative study of pre-menarcheal and menarcheal subjects.. Acta Medica Auxologica..

[14] Hosny LA, El-Ruby MO, Zaki ME, Aglan MS, Zaki MS, El Gammal MA (2005). Assessment of pubertal development in Egyptian girls.. J Pediatr Endocrinol Metab..

[15] Hwang JY, Shin C, Frongillo EA, Shin KR, Jo I (2003). Secular trend in age at menarche for South Korean women born between 1920 and 1986: the Ansan Study.. Ann Hum Biol..

[16] Kaplowitz PB, Slora EJ, Wasserman RC, Pedlow SE, Herman-Giddens ME (2001). Earlier onset of puberty in girls: relation to increased body mass index and race.. Pediatrics..

[17] Goon Daniel T, Toriola Abel L, Uever Jonathan, Wuam Sarah, Toriola Olutoyin M (2010). Growth status and menarcheal age among adolescent school girls in Wannune, Benue State, Nigeria.. BMC pediatrics..

[18] (2006). Directory of Pre-primary, Primary, Junior and Senior Secondary Institutions in Edo State..

[19] Ogunlesi AT, Dedeke IOF, Kuponiyi OT (2008). Socio-economic classification of children attending specialist paediatric centres in Ogun State, Nigeria.. Nigerian Medical Practitioner..

[20] Marfell-Jones Tim Olds Mike, Stewart Arthur, Carter Lindsay (2006). International standards for anthropometric assessment..

[21] Wilson ME, Fisher J, Chikazawa K, Yoda R, Legendre A, Mook D (2003). Leptin administration increases nocturnal concentrations of luteinizing hormone and growth hormone in juvenile female rhesus monkeys.. J Clin Endocrinol Metab..

[22] Singh D, Zambarano RJ (1997). Offspring sex ratio in women with android body fat distribution.. Hum Biol..

[23] Helm P, Munster K, Schmidt L (1995). Recalled menarche in relation to infertility and adult weight and height.. Acta Obstet Gynecol Scand..

[24] Davison KK, Susman EJ, Birch LL (2003). Percent body fat at age 5 predicts earlier pubertal development among girls at age 9.. Pediatrics..

[25] Onland-Moret NC, Peeters PH, van Gils CH, Clavel-Chapelon F, Key T, Tjonneland A (2005). Age at menarche in relation to adult height: the EPIC study.. Am J Epidemiol..

[26] Ofuya Zuleat Millicent (2008). The age at menarche in Nigerian adolescents from two different socioeconomic classes.. Online Journal Of Health And Allied Sciences..

[27] Momoh MI, Okonkwo C (2009). Age at menarche of school girls in urban Benin City.. Annals of Biomedical Sciences..

[28] Kaltiala-Heino R, Kosunen E, Rimpela M (2003). Pubertal timing, sexual behaviour and self-reported depression in middle adolescence.. J Adolesc..

[29] Maskarinec G, Zhang Y, Takata Y, Pagano I, Shumay DM, Goodman MT (2006). Trends of breast cancer incidence and risk factor prevalence over 25 years.. Breast Cancer Res Treat..

[30] Bavdekar A, Yajnik CS, Fall CH, Bapat S, Pandit AN, Deshpande V (1999). Insulin resistance syndrome in 8-year-old Indian children: small at birth, big at 8 years, or both?. Diabetes..

[31] Tunau KA, Adamu AN, Hassan MA, Ahmed Y, Ekele BA (2012). Age at menarche among school girls in Sokoto, Northern Nigeria.. Ann Afr Med..

